# Applications of Synthetic, Natural, and Waste Fibers in Asphalt Mixtures: A Citation-Based Review

**DOI:** 10.3390/polym15041004

**Published:** 2023-02-17

**Authors:** Adham Mohammed Alnadish, Narinderjit Singh Sawaran Singh, Aawag Mohsen Alawag

**Affiliations:** 1 Department of Civil Engineering, Thamar University, Dhamar 87246, Yemen; 2 Department of Transportation & Geotechnical Engineering, Balochistan Campus, National University of Sciences and Technology (NUST), Quetta 87300, Pakistan; 3 Faculty of Data Science and Information Technology, INTI International University, Persiaran Perdana BBN Putra Nilai, Nilai 71800, Negeri Sembilan, Malaysia; 4 Department of Civil Engineering, Universiti Teknologi PETRONAS, Seri Iskandar 32610, Perak, Malaysia

**Keywords:** asphalt, natural fibers, synthetic fibers, waste fibers, dense asphalt mix, stone mastic asphalt, porous asphalt mix, biodegradability

## Abstract

The utilization of synthetic, natural, and waste fibers in asphalt mixtures is constantly increasing due to the capability of fibers to improve the mechanical performance of asphalt mixes. The combination of fibers in asphalt mixes contributes to ecological sustainability and cost benefits. The objective of this paper is to introduce a citation-based review on the incorporation of synthetic, natural, and waste fibers in bitumen, dense-graded asphalt mix, stone mastic asphalt, and porous asphalt mix. Additionally, this article aims to identify research gaps and provide recommendations for further work. The outputs of this article demonstrated that there has recently been a growing interest in the use of natural and waste fibers in asphalt mixtures. However, more future studies are needed to investigate the performance of fiber-modified stone mastic asphalt and porous asphalt mix in terms of resistance to aging and low-temperature cracking. Furthermore, the period of natural fibers’ biodegradability in asphalt mixtures should be investigated.

## 1. Introduction

Asphalt mixture refers to the combination of bitumen, coarse aggregates, fine aggregates, and filler. Asphalt mixtures can be divided into three categories: dense-graded asphalt mix, stone mastic asphalt (SMA), and porous asphalt mix (open-graded asphalt mix) [1,2]. The dense-graded asphalt mix is the most widespread due to its suitability for all pavement layers and traffic conditions and its reasonable cost. Stone mastic asphalt (SMA) consists of 6–7% bitumen, 70–80% coarse aggregates, 8–12% mineral filler, and 0.3–0.5% fibers. SMA is distinguished by its remarkable durability and high resistance to rutting as compared to other types. The main cons of SMA are its higher cost than the dense-graded asphalt by about 20–25%, longer mixing time, and delays in opening to traffic [3,4]. The porous asphalt mix is designed to be used as a drainage layer that allows runoff and rainfall to penetrate the surface layer to the drainage system. The porous asphalt mix is characterized by its high air void content, smooth surface, less tire splash, decreased runoff, and less energy in manufacturing, while its high cost and weakness in carrying heavy traffic loads are among its drawbacks [5,6]. 

On the other hand, permanent deformation, fatigue cracking, low-temperature cracking, and moisture damage are the major distresses that decrease the lifespan of an asphalt layer. Permanent deformation in an asphalt layer occurs due to densification, lateral flow, and subgrade consolidation. In addition, a heavy load, improper compaction, materials, and design for the asphalt layer cause permanent deformation [7,8,9]. Furthermore, the cyclic stress of traffic loads and structural weakness of an asphalt layer due to improper design or aging result in fatigue cracking, which starts from the bottom of the layer and propagates to the surface. Low-temperature cracking is caused by the shrinkage of the asphalt layer in cold regions [10,11]. Furthermore, moisture damage occurs by the loss of bonding between aggregates and bitumen due to the reaction between adhesion materials and water that penetrates the surface layer through cracks [12,13,14]. 

Accordingly, there is a growing global interest in improving the performance of asphalt layers by modifying bitumen or the asphalt mix to extend their lifespan. Fibers have been the most utilized materials in asphalt layers for many decades due to their capability in boosting the mechanical performance of asphalt layers. Fibers can be of natural origin, such as lignin, bamboo, sisal, kenaf, coconut, banana, and Jute, or manmade, such as polypropylene, polyester, polyacrylonitrile (PAN), carbon, aramid, basalt, glass, steel, and ceramic [15,16,17,18]. Moreover, fibers can be used as a bitumen modifier or mixture modifier [19]. It is reported that modifying bitumen with fibers enhances the physical and rheological properties of bitumen in terms of high-temperature performance (rutting) and intermediate-temperature (fatigue cracking) and low-temperature cracking [20,21,22,23]. Additionally, introducing fibers to an asphalt mix by a dry process (modifying mix) has been documented as an effective approach for improving the performance of the asphalt mix, reducing aging of the asphalt mix, and extending the service life of the asphalt layer [24,25,26,27,28]. The use of natural fibers in stone mastic asphalt (SMA) and porous asphalt mix is widely desirable due to their low cost, availability, and absorbency property, which reduces drain down in asphalt mixes [29,30,31]. Furthermore, it is documented that the mechanical properties of an asphalt mix integrated with reclaimed asphalt pavement are notably enhanced by adding fibers. In addition, utilizing fibers in an asphalt mix is cost effective due to the fibers’ ability to decrease maintenance and extend the lifespan of the asphalt layer [32,33,34,35].

The objective of this article is to review and summarize the current state of utilizing natural, synthetic, and waste fibers in asphalt mixtures. Another aim is to identify research gaps and propose recommendations for future work. 

## 2. Physical, Mechanical, and Thermal Properties of Fibers

The physical, mechanical, and thermal properties of fibers play a major role in determining the intended use of fibers. The fiber density is used to identify the suitable content in asphalt mixes. Moisture content refers to the absorbent property of fibers. The tensile strength of fibers indicates the stress that fibers can carry without failure. Young’s modulus and elongation are used to describe the elastic behavior of fibers. The relationship between Young’s modulus and elongation is linear; as Young’s modulus increases, the elongation decreases. In other words, the higher the Young’s modulus, the lower the elastic and ductile behavior of fibers. Thermal conductivity is the ability of fibers to transfer heat. Glass transition represents the temperature at which fibers change from the glassy state to the rubbery state. Glass transition has a major impact on the behavior of the physical properties of fibers [36,37,38]. 

Table 1 shows the general mechanical properties of natural and synthetic fibers. As can be seen in the table, natural fibers are characterized by low density and high absorption rate. Coconut and palm fibers are more elastic than the other fibers. However, the common utilization of natural fibers in stone mastic asphalt and porous asphalt mix is attributed to the high absorption rate. The absorbency of natural fibers decreases the drain down. Therefore, the use of natural fibers in dense asphalt mixes may increase the bitumen content [29,30,31]. On the other hand, synthetic fibers, i.e., polypropylene, polyester, and PAN, are characterized by low density, high elasticity, and low absorption rate, which make them suitable for dense asphalt mixes. Furthermore, the low melting point of polypropylene fibers indicates that polypropylene fibers are desirable for use as a bitumen modifier in hot mix asphalt. The high Young’s modulus and low elongation of basalt fibers and glass fibers refer to the brittle behavior of these fibers, which may break during compaction [14]. Moreover, the prevalent use of carbon fibers and steel fibers as conductive additives for self-healing and microwave deicing of asphalt mixes is attributed to the high thermal conductivity of these fibers.

## 3. Biodegradability of Natural Fibers

Despite the widespread use of naturals fibers due to their availability, low cost, and desirable mechanical properties, they are subjected to premature decomposition as compared to syntactic fibers due to the components of natural fibers such as cellulose, hemi cellulose, and lignin [46,47,48,49]. Table 2 summarizes the biodegradation periods of natural fibers. The degradation of natural fibers due to biodegradability or thermal degradation may negatively affect the performance of natural-fiber-modified asphalt mixes. This, in turn, leads to a decrease in the asphalt layer lifespan. Therefore, the biodegradability period of natural fibers may play a decisive role in the evaluation of asphalt mixes incorporating natural fibers. 

## 4. Methodology

In this study, the literature review on the applications of fibers in asphalt mixtures was conducted by means of citation analysis. Citation analysis is used to identify the most influential research in a specific field through the number of citations. The higher the citations, the better the impact of the research [50,51]. Citation analysis was conducted using the software VOSviewer. The purpose of VOSviewer is to create a network visualization of the imported bibliometric data by creating connection links between citations and documents (clusters). The connection links ensure that the content of the documents are relevant. However, the size of cluster indicates the number of citation, while the link distance between clusters refers to the citations relatedness between documents. The short distance indicates the strong relatedness, while the long distance implies the less relatedness [52]. Moreover, the number of clusters depend on the resolution parameter. The higher the value of resolution parameter, the higher the number of clusters. In this study, the type of analysis in the VOSviewer tool was citation, while the unit of analysis was documents. Furthermore, the minimum number of document’s citations was set to 0 to ensure that the number of citations does not affect the links between documents, while the resolution parameter was set to 1. In addition, the review was focused on the connected documents, while the unconnected, unrelated, and review documents were neglected. However, bibliometric data were obtained from the Dimensions AI database in the form of CSV. Dimensions AI is a free, accessible database that aims to reveal links between articles and their outputs. It is reported that the Dimensions AI database provides higher coverage than Scopus and Web of Science [53,54,55].

The keywords of “Polypropylene fibers AND asphalt”, “Polyester fibers AND asphalt”, “Basalt fibers AND asphalt “, “Glass fibers AND asphalt”, “Steel fibers AND asphalt”, “Aramid fibers AND asphalt”, “Carbon fibers AND asphalt”, “Polyvinyl alcohol fibers AND asphalt”, “Ceramic fibers AND asphalt”, “PAN fibers AND asphalt”, “Cellulose fibers AND asphalt”, “Bamboo fibers AND asphalt”, “Lignin fibers AND asphalt”, “Kenaf fibers AND asphalt”, “Coconut fibers AND asphalt”, “Palm fibers AND asphalt”, “Sisal fibers AND asphalt”, “Jute fibers AND asphalt”, “Banana fibers AND asphalt”, “PET fibers AND asphalt”, “Tire textile fibers AND asphalt”, and “Metallic fibers AND asphalt” were searched in the titles and abstracts of the Dimensions database. The publication year was set to the last five years (2018–2022) to ensure that the sources are recent. However, the publication year from 2000 to 2022 was also set to study the annual publication trend of utilizing fibers in asphalt mixtures.

## 5. Results and Analysis

### 5.1. Annual Publications

**Figure 1:**
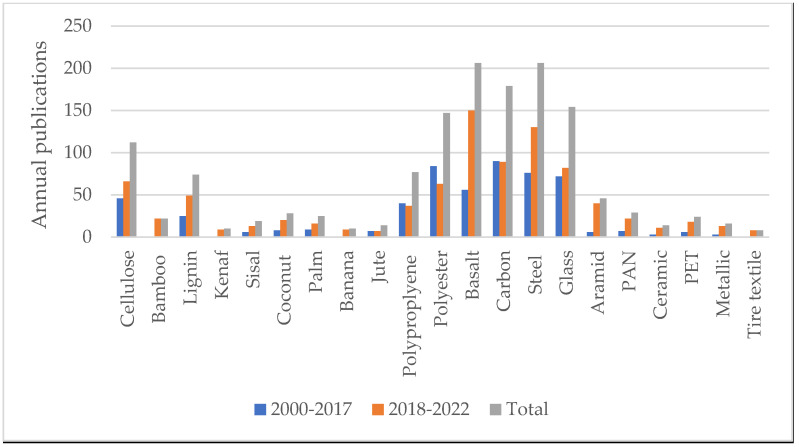
Annual publications.

The annual publications for the applications of fibers in asphalt mixes are shown in Figure 1. It can be seen in the figure that the most utilized fibers in asphalt mixtures are basalt, polyester, carbon, steel, glass, polypropylene, lignin, and cellulose fibers. Moreover, the use of basalt fibers in the years 2018–2022 sharply increased by about 186% as compared to the utilization of basalt fibers from 2000 to 2017. In addition, bamboo, kenaf, coconut, palm banana, jute, aramid ceramic, PET, PAN, metallic, and tire textile fibers have been recently utilized in asphalt mixes. The annual publications of these fibers are located within the last five years. The increase in the use of natural and waste fibers in the last five years indicates the growing interest in this sustainable practice.

### 5.2. Applications of Synthetic Fibers in Asphalt Mixes

#### 5.2.1. Polypropylene Fibers 

**Figure 2:**
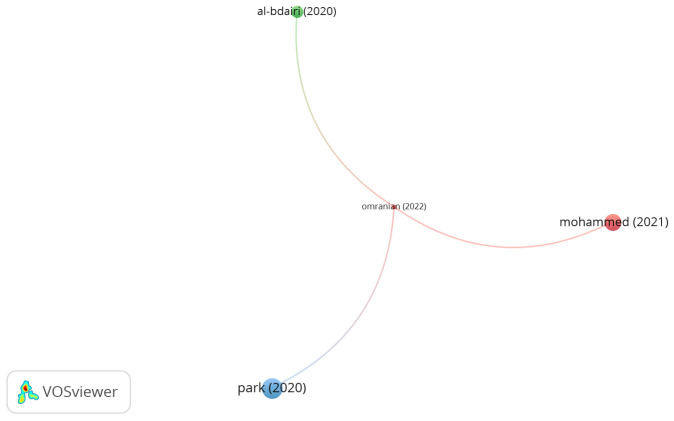
Network visualization of polypropylene fibers.

Polypropylene is widely used as a bitumen modifier due to its low density and melting point of 165 °C. Figure 2 presents the network visualization of documents on utilizing polypropylene in asphalt mixtures. As shown in the figure, there are 3 clusters with 3 links. However, the number of citations for the documents of Park, Mohammed, Al-Badri, and Omaranian were 9, 6, 3, and 0, respectively. While the number of links were 1, 1, 1, and 3 respectively. Park et al. [56] investigated the addition of glass fiber coated with polypropylene into a hot asphalt mix incorporated with reclaimed asphalt pavement. As reported, 10–15 mm long glass fibers improved the tensile strength, moisture resistance, dynamic modulus, and rutting resistance. Mohammed et al. [57] studied amending bitumen with 2, 4, and 6% of polypropylene by bitumen weight. The authors concluded that as the content of polypropylene increases, the tensile strength ratio (TSR) of the warm mix improves. Omaranian et al. [58] reported that the integration of glass and polypropylene fibers at a content of 0.2% by aggregate weight improved the moisture resistance of hot mix asphalt and reduced the cons of freeze and thaw cycles on the performance of the asphalt mix. Al-Badri et al. [59] indicated that the combination of 0.3% steel fibers and 0.1% polypropylene by aggregates weight enhanced the stability and temperature sensitivity of hot mix asphalt. 

#### 5.2.2. Polyester Fibers 

**Figure 3:**
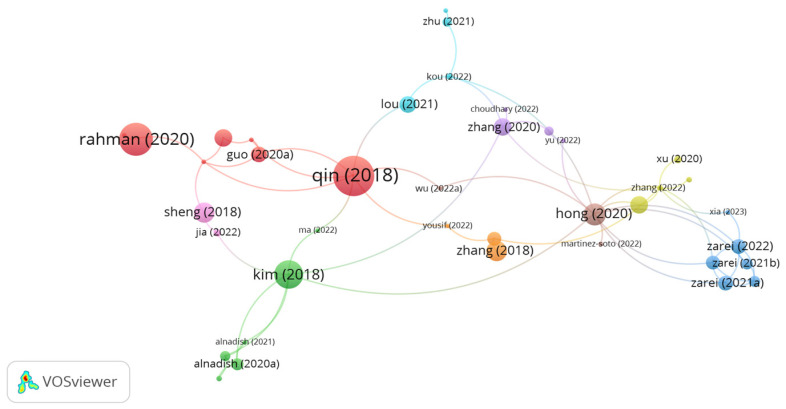
Network visualization of polyester fibers.

Polyester fibers are made with petroleum. The distinctive properties of polyester fibers, i.e., low density, high tensile strength, desirable melting point, and high elasticity, are the major reasons for their widespread use as a bitumen modifier and mixture modifier. Figure 3 shows the most influential documents on utilizing polyester fibers in asphalt mixes based on citation analysis. However, the bigger the size of node, the higher the number of citations, and the greater the impact of document. As seen in the figure, there are 9 clusters with 62 links. The documents of Qin, Kim, Hong, Zhang, and Zarie represents the significant clusters with high number of citations. However, the cluster of Qin incorporating 7 links and 101 citations, while the cluster of Kim containing 7 links and 49 citations. In addition, the document citations number of Hong, Zhang, and Zarie was 30, 26, and 19, respectively. Qin et al. [60] confirmed that modifying a binder with 6 mm long polyester at a content of 5% by bitumen weight enhanced the physical and rheological properties of the binder. Kim et al. [61] assessed hot mix asphalt reinforced by polyester fibers with a length of 6 mm and different contents, 0.5% and 1%, by mix volume. The performance tests showed that adding 1% polyester fibers enhanced the stability, tensile strength ratio (TSR), dynamic stability, and cracking resistance of the asphalt mix. Zhang et al. [62] investigated the use of different fibers, i.e., lignin, polyester, and PAN fibers, in an open-graded asphalt mix. The authors stated that reinforcing the open-graded asphalt mix with polyester fibers at a dosage of 0.3% by mix weight showed the best performance in terms of stiffness modulus, rutting resistance, fatigue resistance, and drainage property in comparison with lignin and PAN fibers. In a similar study, Zhang et al. [63] observed that the integration of 50% steel slag aggregates and 0.45% polyester fibers in a permeable asphalt mix significantly improved the resistance of the mix to low-temperature cracking. Zarie et al. [64,65] evaluated the fracture energy of hot mix asphalt and warm mix asphalt at low and intermediate temperatures. The authors concluded that the addition of 8 mm long polyester fibers at a content of 0.25% by mix weight significantly improved the fracture energy of asphalt mixes. Alnadish et al. [66,67,68] noticed that reinforcing an asphalt mix incorporated with coarse steel slag aggregates with 0.3% polyester fibers enhanced moisture resistance, rutting resistance, cracking resistance, and aging resistance. Hong et al. [69] found that introducing 0.3% polyester fiber to an asphalt mix composed of 0.4% coal gangue powder notably boosted the resistance of the asphalt mix to cracking at low temperatures. In a comparative study conducted by Zhu et al. [70], polyester, basalt, and lignin fibers were separately added at a content of 0.3% by mix weight to an asphalt mix integrated with 40% reclaimed asphalt pavement. The researchers observed that the use of fibers improved the performance of the asphalt mix as compared to the unreinforced mix. Among the fibers, basalt fibers showed the best performance as compared to the reinforced asphalt mix with polyester and lignin fibers. Zhu et al. [71] noticed that reinforcing cold mix asphalt with 0.3% polyester fibers with a length of 6 mm showed better low-temperature performance than the control mix and integrated mix with 0.2% 6 mm long basalt fibers. Yu et al. [72] reported that adding 0.3% polyester fiber to cold asphalt mix increased the resistance of the mix to low temperatures by about 42% in comparison with the control mix. 

#### 5.2.3. Basalt Fibers 

**Figure 4:**
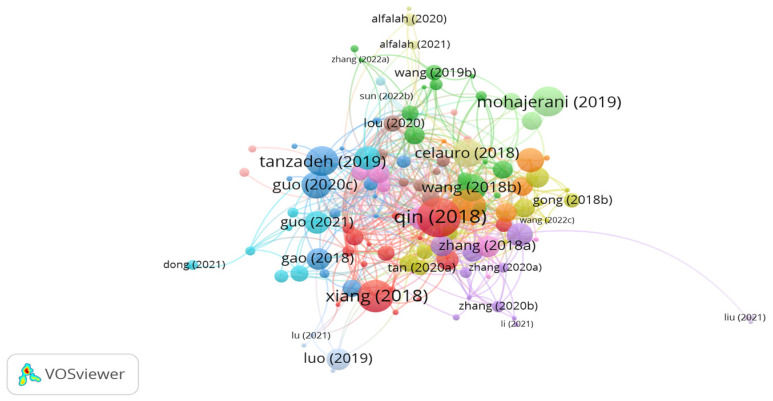
Network visualization of basalt fibers.

Basalt fibers are produced from melted basalt rocks. Influential documents on using basalt fibers in asphalt mixes are shown in Figure 4. It can be seen in the figure that there are 15 clusters with 571 links. The documents of Qin, Xiang, Tanzadeh, Guo, Wang, and Li represents the significant studies with high number of citations. However, the document citations number of Qin, Xiang, Tanzadeh, Guo, Wang, and Li was 101, 67, 56, 46, 63, and 36, respectively. While the number of links was 48, 14, 14, 12, 31, and 26, respectively. Qin et al [60] investigated the use of basalt fibers as binder modifier Qin et al. [60] investigated the use of basalt fibers as a binder modifier. In their study, 6, 9, and 15 mm long basalt fibers were added into base bitumen at different contents of 3, 5, 7, and 10% by bitumen weight. The findings of the study demonstrated that the amended bitumen with 6 mm basalt fibers at doses of 5% and 7% exhibited better performance than the modified binder with lignin and polyester fibers. The authors stated that adding basalt fibers at a content of 10% reduced the force distribution homogeneity and the bonding property between the binder and fibers, while introducing fibers at 3% did not provide a stable network. Tanzadeh et al. [73] investigated the use of 24 mm basalt fibers and 12 mm glass fibers in a porous asphalt mix. The study outputs demonstrated that the inclusion of glass fibers at a content of 0.2% showed better performance than basalt fibers in terms of tensile strength, TSR, Cantabro, and reducing drain down. In addition, the authors noticed that using fibers decreased the permeability of the porous asphalt mix. To overcome the negative effect of fibers in terms of decreasing permeability, the authors introduced nanosilica to the fiber-modified porous asphalt mix. Guo et al. [74] studied the performance of the low-temperature fracture energy of an asphalt mix reinforced separately with different fibers, i.e., basalt, glass, and steel, with various lengths of 6, 12, and 20 mm at a content of 0.5% by mix weight. The study results demonstrated that 6 mm long basalt fibers showed the highest ultimate stain and fracture energy followed by 20 mm long basalt fibers, 6 mm long glass fibers, and 6 mm long steel fibers. The impact of the glass fibers’ length was not significant on the strain. Guo et al. [75] noticed that the addition of basalt fibers to SMA improved cracking resistance and skid resistance. Conversely, adding basalt fibers negatively affects the high-temperature performance and moisture sensitivity. the researchers stated that the combination of lignin fibers and basalt fibers in SMA showed the best performance regarding cracking resistance and rutting resistance. Wang et al. [76,77,78] conducted studies utilizing 6 mm long basalt fibers in hot mix asphalt, asphalt mix incorporated with steel slag, and modified bitumen. The authors concluded that introducing 0.3% basalt fibers by aggregate weight to an asphalt mix containing waste rubber enhanced Marshall stability and the properties of vibration absorption. In addition, the researchers found that hot mix asphalt with basalt fiber displayed improved tensile strength and resistance to freeze–thaw cycles. Furthermore, the authors noticed that the addition of 0.35% basalt fiber to the asphalt mix composed of steel slag aggregates showed the best performance as compared to polyester fibers and lignin with regard to integrity, moisture resistance, and reducing the negative impact of freezing–thawing. In another study conducted by Wang et al. [79], the performance of a porous asphalt mix containing 9 mm long basalt fibers was studied. The findings of the study showed that introducing basalt fibers at a content of 0.3% significantly improved the mechanical properties of the porous asphalt mix in terms of cracking at low temperatures, dynamic stability, Cantabro abrasion, and tensile strength. Xiang et al. [80] concluded that modifying the basalt fiber surface with a solution of silane coupling agent resulted in a rough surface, better compatibility, higher bonding of basalt-modified bitumen, and superior performance of the asphalt mix. According to Zhang et al. [81], the use of 0.3% basalt fibers improves the rheological properties of the binder, stiffness modulus, and creep behavior of the asphalt mix. Gong et al. [82] found that the use of nano TiO2/CaCO3 and basalt fibers enhanced the performance of the asphalt mix after freeze-thaw cycles regarding uniaxial compression static, stability, tensile strength, resilient modulus, and dynamic creep rate. Yoo et al. [83] demonstrated that adding 0.4% basalt fibers into a porous asphalt mix improves tensile strength, rutting resistance, and stability and reduces freezing–thawing effects. Li et al. [84] stated that the incorporation of 9 mm long basalt fibers at a content of 0.4% improved the low-temperature performance. Kong et al. [85] noticed that amending cold mix asphalt with 0.3% basalt fibers notably enhanced its strength and crack resistance. Alfalah et al. [86] indicated that reinforcing an asphalt mix with 0.3% basalt fibers improved the mix’s resistance to rutting. However, the authors indicated that the addition of bitumen negatively affected the rutting performance of asphalt mix. 

#### 5.2.4. Glass Fibers

**Figure 5:**
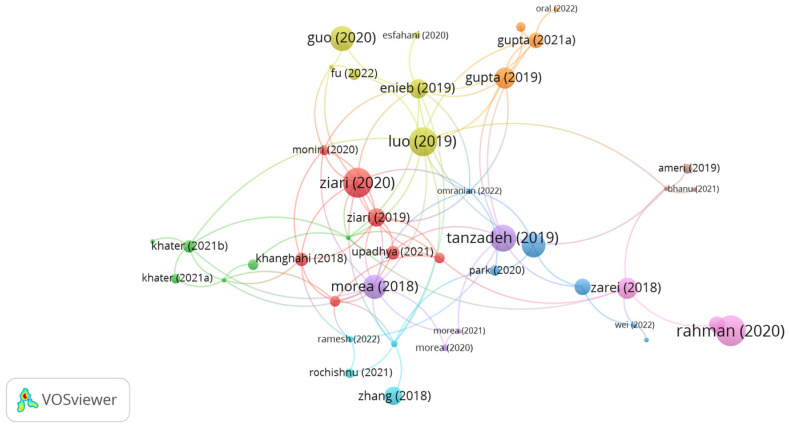
Network visualization of glass fibers.

The influential documents on utilizing glass fibers in asphalt mixtures are shown in Figure 5. A bigger circle size in the network map indicates the stronger impact of the study. As shown in the figure, there are 9 clusters with 94 links. The documents of Tanzadeh, Zairi, Morea, Gupta, and Enieb represents the significant clusters with high number of citations. However, the author’s document citations of Zairi, Tanzadeh, Shanbara, Gupta, Khater, Ramesh, Enieb, Morea, Khanghahi, Guo, Liu and Fu were 65, 56, 44, 19, 8, 4, 27, 44, 15, 46, 21 and 10, while the links were 10, 7, 4, 6, 2, 4, 10, 11, 6, 2, 3 and 2, respectively. Zairi [87] evaluated the low-temperature performance of an asphalt mix containing 12 mm long glass fibers at different proportions of 0.06, 0.12, and 0.18% by mix weight and various contents of reclaimed asphalt pavement, i.e., 25, 50, 75, and 100%. The authors concluded that inserting 0.12% glass fibers notably boosted the low-temperature cracking resistance of the asphalt mix. The authors claimed that introducing glass fibers can improve the performance of an asphalt mix incorporated with 100% reclaimed asphalt pavement. Tanzadeh et al. [73] investigated the use of 0.2% glass fibers, 0.2% basalt fibers, 2% nanosilica, and 4% SBS in a porous asphalt mix. The findings of the study showed that reinforcing the porous asphalt mix with basalt fibers led to the best performance in terms of decreasing drain down. However, nanosilica successfully reduced the sensitivity of the asphalt mix to aging. In similar content, Guo et al. [74] concluded that reinforcing asphalt mix with 6 mm long glass fibers at the content of 0.5% significantly improved the low-temperature performance of asphalt mix. Liu et al. [88] confirmed that treating glass fibers with 2 mol/ L of etchant notably enhanced the fracture energy and the adhesion between glass fibers and emulsified asphalt. Shanbara et al. [89] investigated the impact of adding glass and hemp fiber into cold asphalt mix. The study findings elucidated that glass fibers with 14 mm length and 0.35% content by aggregate weight along with hemp fiber considerably enhanced cracking and rutting resistance. In a comparative study, Gupta [90] investigated the feasible incorporation of different fibers, i.e., 0.05% aramid fibers, 0.05% aramid pulp, 0.5% cellulose fibers, and 0.5% hybrid glass fibers (cellulose fibers + glass fibers) in a porous asphalt mix. The authors corroborated that the utilization of fibers in the porous asphalt mix successfully reduced drain down and improved indirect tensile strength (ITS). The integrated mixture with aramid pulp showed the best performance, followed by hybrid glass fibers, aramid fibers, and cellulose fibers. Khater et al. [91] studied the potential use of 12 mm long glass and lignin fibers in an asphalt mix. The study outputs demonstrated that the incorporation of glass and lignin fibers in the asphalt mix at a content of 0.3% by mix weight enhanced moisture resistance and low-temperature cracking resistance. Ramesh et al. [92] noticed that adding 0.3% nanoglass fiber by mix weight into warm mix asphalt composed of 70% reclaimed asphalt pavement improved fracture energy by about 1.8 times. Enieb et al. [93] evaluated the performance of an asphalt mix incorporated with 6 mm and 12 mm long glass fibers in terms of indirect tensile strength, moisture sensitivity, fatigue cracking, resilient modulus, and fracture energy. The authors indicated that adding 6 mm long glass fibers to the asphalt mix led to a positive impact in terms of improving the mechanical properties and aging resistance of the asphalt mix. According to Morea et al. [94], introducing 36 mm long glass fibers to hot mix asphalt at a dosage of 0.4% showed better fatigue resistance and permeant deformation resistance than the control mix, reinforced mix with 25 mm long polyester, and modified mix with 12 mm long glass fibers. Khanghahi et al. [95] noticed an increase in fracture energy at low temperatures for hot asphalt mix integrated with 6% gilsonite by bitumen weight and 12 mm long glass fibers at a content of 0.3% by mix weight. Mohammed et al. [96] concluded that modifying the binder with 12 mm long glass fibers at a proportion of 1% by bitumen volume led to better performance than the unmodified bitumen and modified bitumen with cellulose fibers in terms of cracking and rutting resistance. Fu et al. [97] evaluated an amended asphalt mix with glass fiber at the optimum content of 0.5% by mix weight. The outputs of the study emphasized that adding glass fiber enhanced the tensile strength and cracking resistance of the asphalt mix.

#### 5.2.5. Steel Fibers

**Figure 6:**
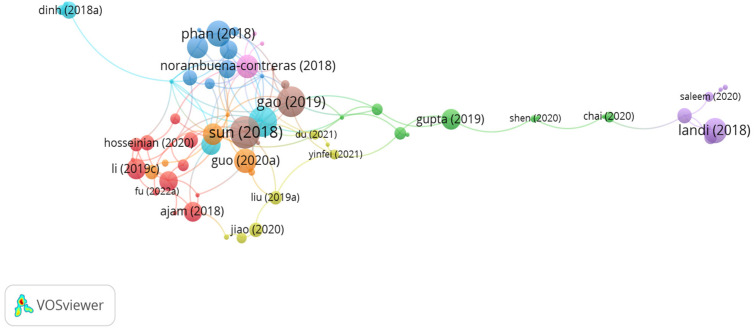
Network visualization of steel fibers.

According to the citation analysis, influential documents on the applications of steel fibers in asphalt mixes were identified using the VOSviewer tool. Figure 6 demonstrates the network connections between documents and citations. As seen in the figure, there are 9 clusters with 131 links. The significant clusters with high number of citations are represented by the studies of Liu, Phan, Gao, Tabaković, González, Yang, Jiao, Li, Dinh, Hosseinian, and Norambuena-Contreras. However, the citations for the document of Liu, Phan, Gao, Tabaković, González, Yang, Jiao, Li, Dinh, Hosseinian, and Norambuena-Contreras were 15, 53, 75, 11, 29, 7, 17, 15, 40, 19, and 45, respectively. While the number of links was 4, 6, 8, 4, 7, 3, 2, 4, 4, 4, and 12, respectively. Liu et al. [98] introduced steel fibers into the binder at various contents of 2, 3, and 4% by bitumen weight and different lengths, i.e., 1 mm–7 mm in 2 mm increments, to study healing efficiency. The findings of the study revealed that amending the binder with 5 mm long steel fibers at a content of 4% led to the best performance regarding healing efficiency and fatigue life recovery, while the dosage of 3% led to the worst performance. Phan et al. [99] studied the potential use of steel wool fibers and steel slag aggregates in an asphalt mix as self-healing additives using microwave heating. The authors concluded that the use of 2% steel wool fibers by binder weight and 30% of coarse steel slag aggregate significantly enhanced the healing efficiency of the asphalt mix. Similarly, Gao et al. [100] assessed microwave deicing of an asphalt mix incorporated with different sizes of steel wool fibers: 0.015–0.035, 0.050–0.070, and 0.075–0.125 mm. The authors pointed out that the ice thawing time for steel wool fibers (0.015–0.035) at a content of 0.3%, steel wool fibers (0.050–0.070) at a proportion of 0.6%, and steel wool fibers (0.075–0.125) at a dosage of 0.9% were 9.3, 11.3, and 14.8%, respectively. In another study conducted by Tabaković et al. [101], the self-healing property of a porous asphalt mix containing steel wool fibers 10 mm long and 0.09 mm in diameter was investigated. The results of the study indicated that the addition of steel wool fibers at the proportions of 10% and 15% by binder weight led to lower strength recovery than the amended bitumen with 5% steel wool fibers. González et al. [102] investigated microwave crack healing on an asphalt mix incorporated with different additives, i.e., 4% steel wool fibers, 4% metal shaving, and 5.3% silicon carbide, by bitumen volume. The findings of the study showed that there was a small reduction in the average healing ratio of cracks in the asphalt mix. The authors also stated that heating natural aggregates by microwave could heal cracks without additives. According to Yang et al. [103], utilizing steel fibers in an asphalt mix led to better fracture behavior, moisture resistance, crack healing ability, and less sensitivity to freeze–thaw cycles than the modified asphalt mix with steel wool fibers and carbon fibers. Furthermore, Jiao [104] investigated the low-temperature fracture resistance of an asphalt mix reinforced with steel fibers and basalt fibers. The authors noticed that the asphalt mix containing 6 mm long basalt fibers showed desirable ductal behavior, while the amended mix with 6 mm or 12 mm long steel fibers demonstrated brittle characteristics. Li et al. [105] observed that the healing crack reached 92.3% by modifying the asphalt mix with steel wool fibers at a dosage of 6% by bitumen volume. Additionally, after induction heating, the air void content in the modified asphalt mix was reduced by about 17%. Similarly, Dinh et al. [106] noticed that the combination of steel wool fibers and steel slag aggregates exhibited a higher healing rate than the mixtures consisting of steel wool fibers and granite aggregates. In addition, Dinh et al. [107] observed in another study that the use of reclaimed asphalt pavement in an asphalt mix reinforced with steel wool fibers decreased the efficiency of induction healing due to aging and oxidation processes. As a result, the authors suggested the use of a cooking oil/rejuvenator to enhance induction heating and the healing rate. Hosseinian et al. [108] investigated the moisture resistance and conductivity of an asphalt mix reinforced with different proportions of steel wool fibers in the range of 2–10% in 2% increments by binder volume. The authors concluded that introducing 6% steel wool fibers improved the indirect tensile strength and tensile strength ratio of the asphalt mix. Norambuena-Contreras et al. [109] indicated that adding steel wool fibers and metallic waste enhanced the conductivity of the asphalt mix but not steel shavings. However, a decrease in thermal conductivity was observed in the asphalt mix modified with metallic waste. 

#### 5.2.6. Carbon Fibers 

**Figure 7:**
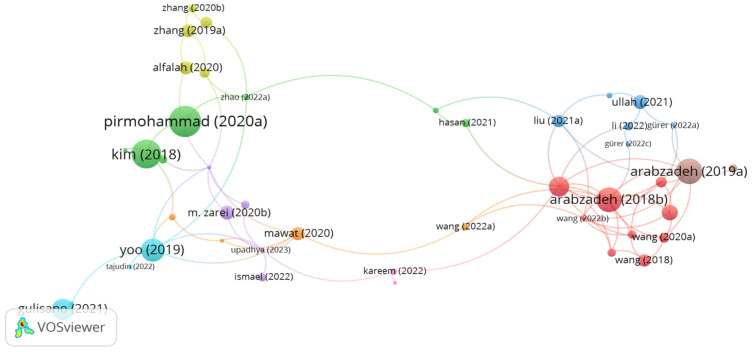
Network visualization of carbon fibers.

The components of carbon fibers are 90% PAN and 10% rayon. The network map of connections between documents and citations on using carbon fibers in asphalt mixes is displayed in Figure 7. As can be seen in the figure, there are 9 clusters with 82 links. The documents of Arabzadeh, Pirmohammad, Kim, Yoo, and Ullah represents the most significant clusters. However, the document citations of Arabzadeh, Pirmohammad, Kim, Yoo, Zhang, Ismael, Gürer, and Ullah were 37, 56, 49, 32, 10, 4, and 12, respectively. Whereas the number of links was 9, 2, 4, 7, 4, and 3, respectively. Arabzadeh et al. [110] studied the use of carbon fibers as a conductive additive in an asphalt mix. The study outputs demonstrated that inserting 0.5% carbon fibers enhanced the conductive property of the asphalt mix. Pirmohammad et al. [111] evaluated the low-temperature fracture performance of an asphalt mix reinforced separately with 4, 8, and 12 mm long carbon and kenaf fibers at different dosages of 0.1, 0.2, and 0.3% by mix weight. The study results demonstrated that adding 8 mm kenaf fibers and 4 mm carbon fibers at a content of 0.3% effectively enhanced the fracture resistance of the asphalt mix at low temperatures. Kim et al. [61] stated that reinforcing an asphalt mix with 6 mm long carbon fibers at a content of 1% by mix volume led to better cracking resistance, permeant deformation resistance, and moisture resistance than the unreinforced asphalt mix. In a study conducted by Yoo et al. [112], the healing property of an asphalt mix modified by carbon fibers and graphite nanofibers was studied. The authors asserted that the incorporation of fibers at a proportion of 0.5% considerably improved the efficiency of healing. The researchers stated that the recovery improved by 40% as compared to the control mix. Zhang et al. [113,114] concluded that inserting 0.05% cured carbon fibers by mix weight to a porous asphalt mix significantly improved indirect tensile strength and rutting resistance. In addition, at the optimum fiber content of 0.15%, the infiltration and porosity of the asphalt mix decreased by about 20% and 7%, respectively. Moreover, the authors stated that with the increase in fiber dosage, the ductile behavior and cracking resistance of the asphalt mix improved. Ismael et al. [115] investigated the utilization of carbon and jute fibers in SMA. The outputs of the study demonstrated that adding 7.5 mm long carbon or jute fibers at a proportion of 0.5% by mix weight notably enhanced Marshall stability and rutting resistance and decreased drain down of the asphalt mix. According to Gürer et al. [116,117], adding 5 mm long carbon fibers with a length of 5 mm and content of 0.2% by mix mass into hot mix asphalt and SMA improved the conductive property of the asphalt mix. The researchers also stated that the gradation of the asphalt mix has a significant effect on the conductive property. The denser the gradation, the higher the conductivity. Schuster et al. [118] concluded that the integration of 1% carbon fiber and 16% steel wool fiber by bitumen weight significantly improved the healing rate. Similarity, Ullah et al. [119] indicated that the incorporation of 0.2–0.4% carbon fiber by mix weight in an asphalt mix remarkably enhanced the conductivity and performance of the asphalt mix.

#### 5.2.7. Aramid Fibers

**Figure 8:**
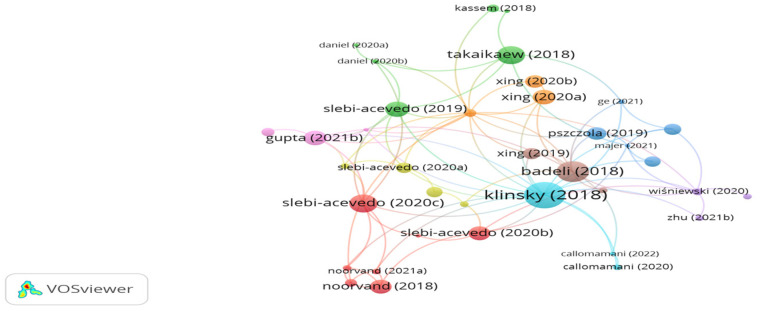
Network visualization of aramid fibers.

Aramid fibers are produced from aromatic polyamides. The network map shown in Figure 8 demonstrates the citation analysis outputs. It is shown in the figure that there are 9 clusters with 85 links. However, the study’s citations of Takaikaew, Klinsky, Slebi-Acevedo, Callomamani, Hajiloo, Gupta, Noorvand, Daniel, and Xing were 30, 62, 30, 3, 10, 6, 3, 2, and 11, respectively. While the number of links was 6, 18, 8, 2, 2, 16, 4, 5, and 3, respectively. Takaikaew et al. [120] studied the performance of hot mix asphalt reinforced with 19 mm long polyolefin-aramid fibers at a dosage of 0.05% by mix weight. The study findings illustrated that adding fibers significantly improved the resilient modulus, tensile strength, fatigue resistance, and rutting resistance. Similarly, Klinsky et al. [121] indicated that the addition of polypropylene-aramid enhanced the mechanical properties of an asphalt mix. According to the findings obtained by Slebi-Acevedo et al. [122], adding 19 mm long polyolefin-aramid or 4 mm long PAN at a content of 0.3% by mix weight improves the low-temperature performance of an asphalt mix regarding fracture energy and cracking resistance. Additionally, Callomamani et al. [123] found that introducing 0.065% aramid or 0.1% polyethylene terephthalate (PET) or 0.065% PAN fibers to hot asphalt mix boosted the rutting resistance, cracking resistance, and tensile strength of the asphalt mix. Hajiloo et al. [124] studied the performance of hot mix asphalt reinforced with different proportions of polyolefin-aramid fibers, i.e., 0.025, 0.05, and 0.1%, by mix weight. The researchers noticed that the higher the content of fibers, the better the fracture toughness. Gupta et al. [125] stated that reinforcing a porous asphalt mix with 12 mm long aramid fibers notably boosted the tensile strength, moisture resistance, and cracking resistance of the asphalt mix. Xing et al. [126] confirmed that modifying bitumen with 2 mm long aramid fibers at a proportion of 2% by bitumen weight improved rheological and physical properties at high and low temperatures. According to Noorvand [127], adding 19 mm long aramid fibers at a dosage of 0.05% by mix weight to hot mix asphalt incorporated with 20% reclaimed asphalt pavement enhanced fatigue and cracking resistance. Moreover, 19 mm long aramid fibers exhibited the best performance in comparison with 10 mm and 38 mm long aramid fibers. Additionally, Daniel et al. [128] found that introducing 19 mm long polyolefin-aramid fibers into warm mix asphalt improved stiffness modulus by 89% and fatigue life by 100%. Slebi-Acevedo et al. [129] concluded that the combination of a porous asphalt mix with 19 mm polyolefin-aramid fibers at a dosage of 0.05% by mix weight and hydrate lime enhanced the mechanical properties of the porous asphalt mix. 

#### 5.2.8. Polyacrylonitrile (PAN) Fibers

**Figure 9:**
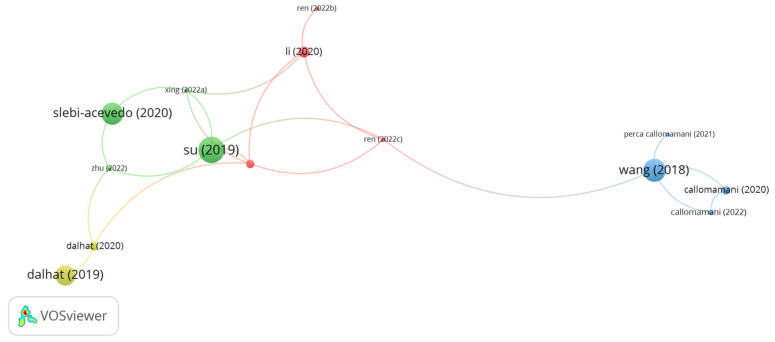
Network visualization of PAN fibers.

Significant studies using PAN fibers in asphalt mixes as reinforcement materials are demonstrated by network visualization shown in Figure 9. As seen in the figure, there are 4 clusters with 20 links. The clusters of Su, Slebi-Acevedo, Dalhat, and Wang exhibited the highest number of citations. However, the research’s citations of Su, Slebi-Acevedo, Dalhat, and Wang were 25, 18, 15, and 20, respectively. Su et al. [130] investigated amending bitumen with 3% PAN fiber and modified PAN fiber with self-polymerization of dopamine and covalent grafting. The study results demonstrated that the amended bitumen with modified PAN fibers increased the adhesion property and moisture resistance by 8% as compared to the unmodified PAN fiber. Slebi-Acevedo et al. [131] affirmed that the addition of 6 mm long PAN fibers to a reclaimed asphalt mix at a content of 0.3% by mix weight enhanced the mechanical performance of the asphalt mix up to 50% without the use of rejuvenators. Dalhat et al. [132] noticed a significant enhancement in the dynamic modulus, rutting resistance, and cracking resistance of an asphalt binder modified with 1% PAN fiber and 1% SBS. According to Xing et al. [133], blending base bitumen with SBS and PAN fibers surface-modified by bionic coating and nanosilica improves the rheological properties and shear strength of bitumen. Wang et al. [134] indicated that reinforcing an asphalt mix with 6 mm long PAN at a dosage of 0.3% by mix weight boosted the fatigue resistance of the asphalt mix. 

#### 5.2.9. Ceramic Fibers

**Figure 10:**
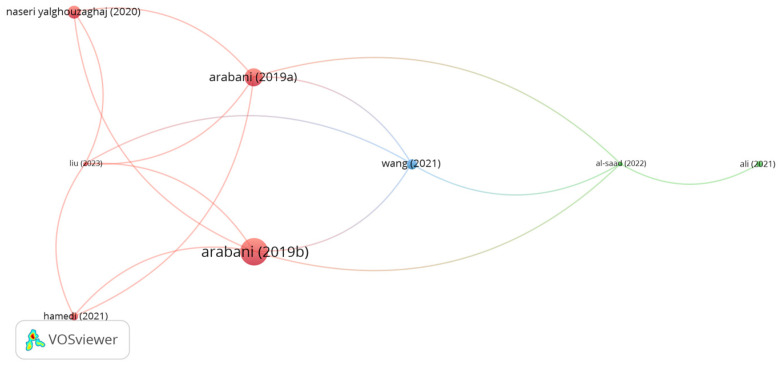
Network visualization of ceramic fibers.

Recently, there is a growing use of ceramic fibers as bitumen fibers. Influential studies using ceramic fibers in asphalt mixes are shown in Figure 10. It is shown in the figure that the cluster of Arabani, Naseri, Wang, and Hamedi exhibited the highest number of citations. However, the author’s document of Arabani, Naseri, Wang, and Hamedi was 35, 8, 5, and 3, respectively. Arabani et al. [135,136] confirmed that the combination of 20 mm long ceramic fibers at a dosage of 3% by bitumen weight into the base binder enhanced the high-temperature performance and decreased the performance of the binder at low temperatures. On the contrary, Naseri et al. [137] stated that modifying bitumen with 0.4% ceramic fibers by bitumen weight boosted the fracture energy and toughness of the binder, which, in turn, decreased cracking at low temperatures. Wang et al. [138] noticed the presumed increase in moisture resistance, rutting resistance, and resistance to low-temperature cracking of an asphalt mix reinforced with 2–4 mm long ceramic at a proportion of 0.4% by mix weight. Hamedi et al. [139] concluded that modifying bitumen with 3% ceramic fibers improved the resistance of SMA to rutting. In a study conducted by Liu et al. [140], an asphalt mix was modified with ceramic fibers at a dosage of 0.3% by mix mass to study the influence of adding ceramic fiber on the properties of the binder. The findings of the study illustrated that the addition of ceramic fibers improved the freeze–thaw splitting and moisture resistance of the asphalt mix. 

### 5.3. Applications of Natural Fibers in Asphalt Mixes 

#### 5.3.1. Cellulose Fibers

**Figure 11:**
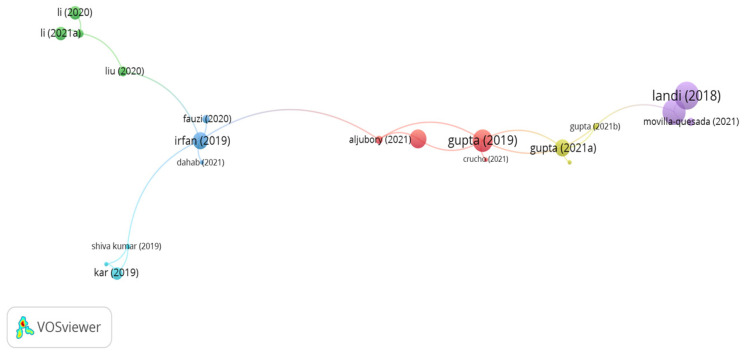
Network visualization of cellulose fibers.

Wood and paper industries and the byproducts of plants are the main sources of cellulose fibers. The outputs of the citation analysis are illustrated in Figure 11. A bigger size of the node implies a higher impact of the document, while the links describe the citation connections among the documents. It can be seen in the figure that there are 6 clusters with 22 links. In addition, the significant clusters with high number of citations are belong to the documents of Landi, Irfan, Fauzi, and Li. However, the citations for the document of Landi, Irfan, Fauzi, and Li were 50, 20, 5, and 13, respectively. Landi et al. [141,142] evaluated a porous asphalt mix incorporated with 0.3% cellulose fibers and 0.3% end-of-life tire fibers by mix weight. The study findings demonstrated that both fibers showed better fatigue resistance than the unreinforced asphalt mix. However, the asphalt mix incorporated with end-of-life tire fibers displayed higher fatigue resistance than the amended mix by about 70%. The researchers also found that the cumulative energy demand and global warming potential of reinforced porous asphalt with end-of-life tire decreased by 25% and 10%, respectively, in comparison with porous asphalt composed of cellulose fibers. Irfan et al. [143] found that adding 0.3% cellulose fibers to stone mastic asphalt significantly enhanced rutting and fatigue resistance. In similar content, Fauzi et al. [144] confirmed that introducing 0.3% cellulose fibers to stone mastic asphalt improved the stiffness modulus. According to Li et al. [33], aging has a negative impact on cellulose fibers; aging resulted in a decrease in the cracking resistance of an asphalt mix containing cellulose fibers. 

#### 5.3.2. Bamboo Fibers 

**Figure 12:**
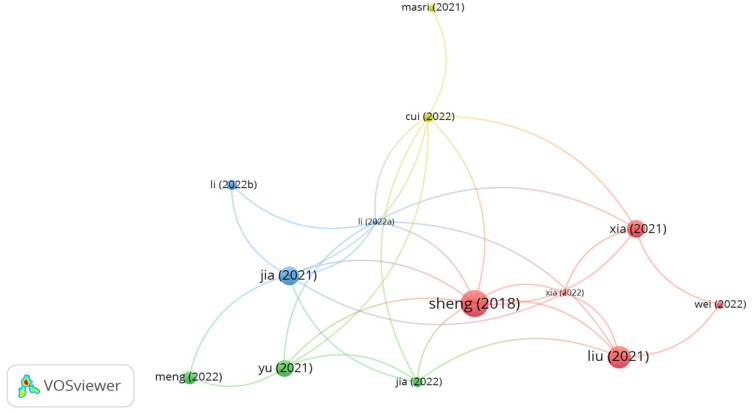
Network visualization of bamboo fibers.

Influential studies on the applications of bamboo fibers in asphalt mixes are shown in Figure 12. As seen in the figure, the document of Sheng, Liu, Xia, Jia, and Meng displayed bigger size of node than the other documents. However, the document citations of Sheng, Liu, Xia, Jia, and Meng were 26, 17, 11, 12, and 6, respectively. Sheng et al. [145] assessed the potential use of bamboo, lignin, and polyester fibers in a dense asphalt mix and stone mastic asphalt mix. The study concluded that adding 0.2% bamboo fibers to dense asphalt and 0.4% to stone mastic asphalt led to better performance than the control mix and modified mix with polyester and lignin fibers. Similarly, Liu et al. [146] investigated the performance of asphalt containing 0.4% lignin and 0.3% bamboo fibers. The findings of the study illustrated that both fibers improved the mechanical properties of the asphalt mix regarding low-temperature performance, high-temperature performance, and moisture sensitivity. However, the researchers stated that bamboo fibers notably boosted the thermal properties of the asphalt mix. Furthermore, the authors noticed that adding bamboo and lignin fibers enhanced the performance of asphalt mixes subjected to long-term aging. Xia et al. [147,148] evaluated the performance of SMA and hot mix asphalt incorporated with lignin and bamboo fibers. The authors noticed that the durability of SMA was better than dense hot mix asphalt. However, the positive effect of utilizing lignin and bamboo on freeze–thaw was almost the same. The researchers stated that the incorporation of bamboo fibers in the asphalt mix led to better aging performance than the modified mix with lignin fibers. Based on the findings obtained by Jia et al. [149], the inclusion of bamboo fibers in an asphalt mix at a proportion of 0.3% by mix weight remarkably improved the rutting and fatigue cracking resistance of the asphalt mix. Meng et al. [150] found that amending bitumen with bamboo fibers, SBS, and soybean bio-asphalt at a dosage of 3% by bitumen weight led to better performance than the modified bitumen with 5% SBS in terms of rheological properties. 

#### 5.3.3. Palm Fibers

**Figure 13:**
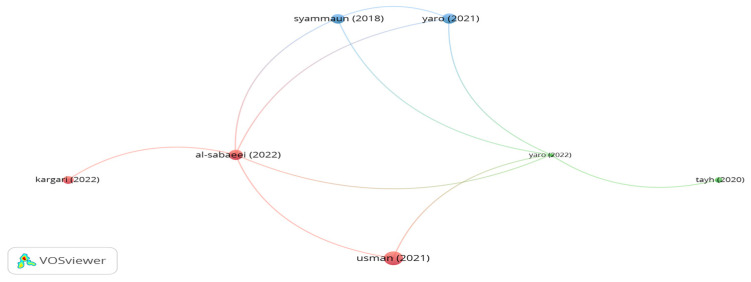
Network visualization of palm fibers.

The outputs of citation analysis by means of the VOSviewer tool for impactful studies using palm fibers as reinforcement materials are illustrated in Figure 13. It is seen in the figure that there are 3 clusters with 11 links. However, the citations number of the significant documents of Syammaun, Yaro and Tayh was 6, 7, and 2, respectively. Syammaun et al. [151] added palm oil fibers by a dry process at different proportions in the range of 1–5% in 1% increments by bitumen weight to investigate the influence of palm fibers on the resilient modulus of a porous asphalt mix. The authors noticed that adding palm fibers at 3% led to the best performance. Yaro et al. [152,153] concluded that the use of sequential mixing for palm oil fibers in stone mastic asphalt enhanced the moisture resistance, stiffness modulus, Cantabro, and drain down of SMA. It is reported that the optimum content of palm fiber was 0.3% by mix weight. Tayh et al. [154] indicated that modifying bitumen with 0.75% palm fibers by binder weight enhances the physical properties and rutting resistance of bitumen. 

#### 5.3.4. Lignin Fibers 

**Figure 14:**
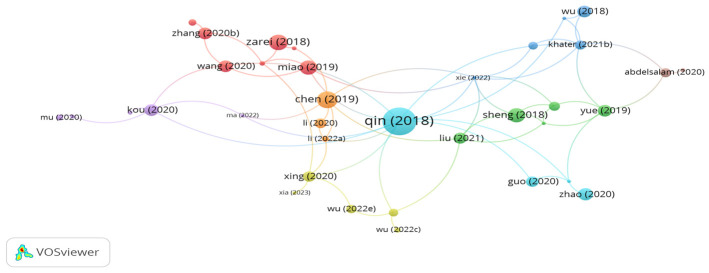
Network visualization of lignin fibers.

Wood pulp, cotton, hemp, jute, and paper industries are the sources of lignin. Figure 14 displays influential documents based on the conducted citation analysis. It can be seen in the figure, that there are 9 clusters with 74 links. However, the document of Qin, Chen, Wang, Wu, and Kou exhibited higher number of citations than the other documents. The document citations number of Qin, Chen, Wang, Wu, and Kou was 101, 38, 19, and 18, respectively. Qin et al. [60] investigated the rheological and physical properties of modified bitumen with lignin, polyester, and basalt fibers. The study findings demonstrated that the addition of basalt fibers at contents of 5% and 7% showed the best performance, followed by lignin fibers at a dosage of 3% by bitumen weight. Wang et al. [77] observed that introducing lignin fibers at a dosage of 0.23% by mix weight significantly improved the moisture resistance of an asphalt mix incorporated with steel slag aggregates. Chen et al. [155] studied the physical and rheological properties of modified binder with corn stalk, basalt, and lignin fibers. The findings of study demonstrated that modifying binder with stalk fibers showed the best performance, followed by basalt, and lignin fibers. Ma et al. [156] investigated the performance of modified bitumen with different types of fibers, i.e., basalt, lignin, polyester, and composite fibers, at proportions of 7, 7, 7, and 6% by binder weight. The researchers indicated that all fibers boosted the shear strength of the binder as compared to the unmodified binder. However, the amended binder with composite fibers showed superior performance, followed by basalt, lignin, and polyester. In studies conducted by Wu et al. [157,158], the cracking resistance and high-temperature performance of stone mastic asphalt reinforced with 0.3% lignin and 0.3% basalt fibers were assessed. The results of the studies illustrated that the use of fibers improves the performance of the asphalt mix in comparison with the control mix. Moreover, the authors concluded that basalt showed the best performance. According to Kou et al. [159,160], the appropriate contents of basalt fibers, polyester fibers, and lignin fibers as bitumen modifier are 2%, 3%, and 4% by bitumen weight, respectively. The authors confirmed that these contents remarkably enhanced performance in terms of permanent recovery and cracking resistance. 

#### 5.3.5. Coconut Fibers

**Figure 15:**
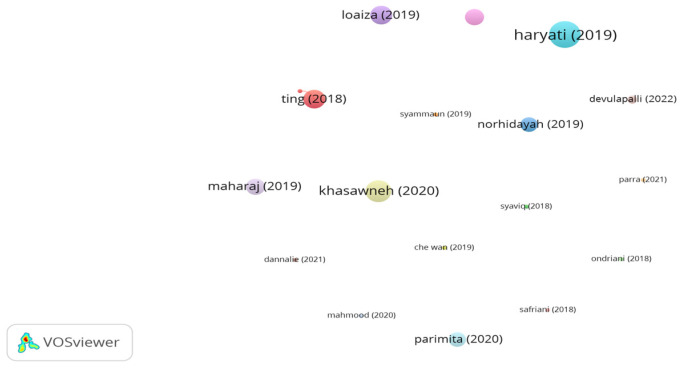
Network visualization of coconut fibers.

Recently, the use of coconut fibers in asphalt mixes has increased. Significant studies utilizing coconut fibers in asphalt mixes are highlighted through a network map of documents and citations in Figure 15. The disappearance of links indicates that there were no linked citations among the documents. Khasawneh et al. [161] noticed that adding coconut fibers at a dosage of 0.4% by aggregate weight improved the stability of an asphalt mix. In addition, the researchers stated that the incorporation of coconut fibers has a significant effect on the volumetric properties of the asphalt mix. Maharaj et al. [162] found that adding 2.5 mm long coconut fibers as a bitumen modifier at a proportion of 6% by binder weight improved the rheological properties of bitumen in terms of deformation resistance and elastic behavior. In a study conducted by Parimita [163], the Marshall stability of stone mastic asphalt reinforced with 0.3% coconut and banana fibers were evaluated. The outputs of the study illustrated that adding coconut/ banana fibers significantly improved Marshall stability of asphalt mix as compared to the unreinforced mix. Norhidayah et al. [164] concluded that introducing 0.3% coconut fibers by mix weight into a porous asphalt mix composed of coconut shells as coarse aggregates reduced the drainage of the mix. In another study conducted by Haryati et al. [165], the potential integration of 10% coconut shell and 0.3% coconut fibers in a porous asphalt mix was assessed. The study findings demonstrated that the addition of coconut shells and fibers notably boosted the rutting resistance and stability of the asphalt mix. 

#### 5.3.6. Sisal Fibers 

**Figure 16:**
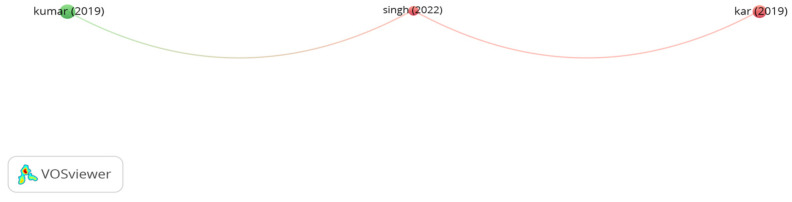
Network visualization of sisal fibers.

The outputs of the citation analysis of influential documents on using sisal fibers are demonstrated in Figure 16. Kar et al. [166] evaluated the feasible use of sisal fibers and fly ash in dense asphalt mix and stone mastic asphalt in terms of Marshall stability. The results of the study indicated that adding 0.3% sisal fibers improved the Marshall stability of asphalt mixes. In a similar study, Kumar [167] observed that the stability and tensile strength of control stone mastic asphalt were almost comparable to stone mastic asphalt containing 0.3% sisal fibers. Singh et al. [168] concluded that using 0.4% sisal fibers, 0.3% coir fibers, and 0.3% rice straw fibers in SMA significantly improved the tensile strength and stability of stone mix, and drain down also decreased. 

#### 5.3.7. Kenaf Fibers

**Figure 17:**
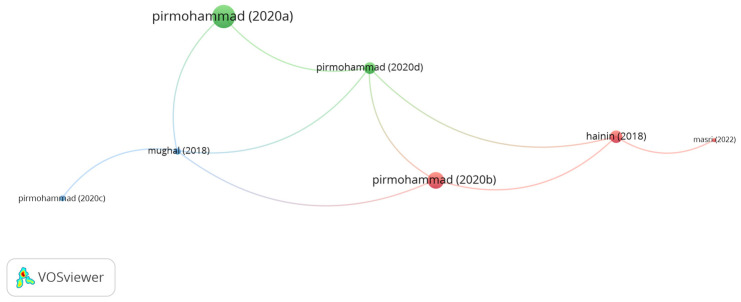
Network visualization of kenaf fibers.

Kenaf fibers are one of the commonly used natural fibers in asphalt mixes. The research on reinforcing asphalt mixtures with kenaf fibers has been reviewed based on the impact of the studies. Figure 17 shows the poignant documents on the use of kenaf fibers. It can be seen in the figure that the number of clusters is 3, while the number of links is 9. However, the documents of Pirmohammad and Hainin showed the highest number of citations as compared to the other documents. Pirmohammad et al. [111] noticed that introducing 8 mm long kenaf fibers at a content of 0.3% into dense asphalt mix enhanced the low-temperature cracking resistance of the asphalt mix. In another study conducted by Pirmohammad et al. [169], the low-temperature performance of asphalt mixes modified with kenaf and goat wool fibers at the proportions of 0.1, 0.2, and 0.3% by mix weight and lengths of 4, 8, and 12 mm was evaluated. The study concluded that the addition of 8 mm long kenaf/4 mm long goat wool fibers significantly improved the fracture energy of the asphalt mix at low-temperature. Hainin et al. [170] confirmed that introducing 30 mm long kenaf fibers to dense asphalt mix enhances the tensile strength ratio and rutting resistance of the asphalt mix. Syafiqah et al. [171] noticed a significant enhancement in the resilient modulus, Marshall stability, and dynamic stability of stone mastic asphalt reinforced with 0.2% kenaf fibers. Masri et al. [172] stated that adding kenaf fibers to a porous asphalt mix at a dosage of 0.6% by mix weight enhanced the resilient modulus of the asphalt mix. 

#### 5.3.8. Jute Fibers

**Figure 18:**
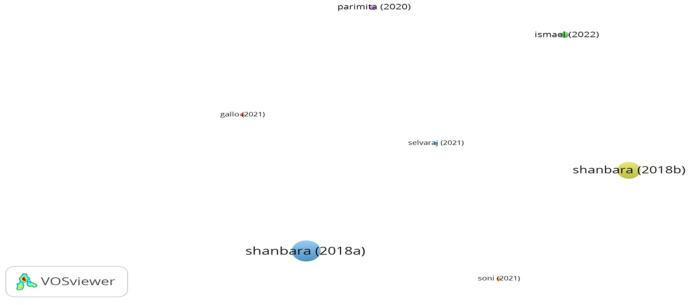
Network visualization of jute fibers.

Influential studies on reinforcing asphalt mixes with jute fibers are displayed in Figure 18. It can be seen in the figure that there are no links between documents. This is because there were no citations between the studies. Selvaraj et al. [173] investigated the interface behavior between coir geotextile, jute geotextile, and asphalt layer by means of numerical analysis. The outputs of study demonstrated that the use of coir geotextile showed better performance than the geotextile of jute in terms of delaying degradation and decreasing developed stress at surface of asphalt layer. According to Shanbara et al. [89,174], the use of 14 mm long jute fibers at a content of 0.35% by mix weight in cold mix asphalt significantly improved the rutting resistance and indirect tensile strength of the asphalt mix. Ismael et al. [115] demonstrated that modifying stone mastic asphalt with 7.5 mm long jute fibers at a dosage of 0.5% by mixture mass notably enhanced the rutting resistance and dynamic stability and decreased drain down of SMA. Gallo et al. [175] confirmed that the addition of 20 mm long jute fibers at the content of 0.2% into an asphalt mix increased air voids content, resilient modulus, and indirect tensile strength, while the tensile strength ratio slightly decreased. 

#### 5.3.9. Banana Fibers 

**Figure 19:**
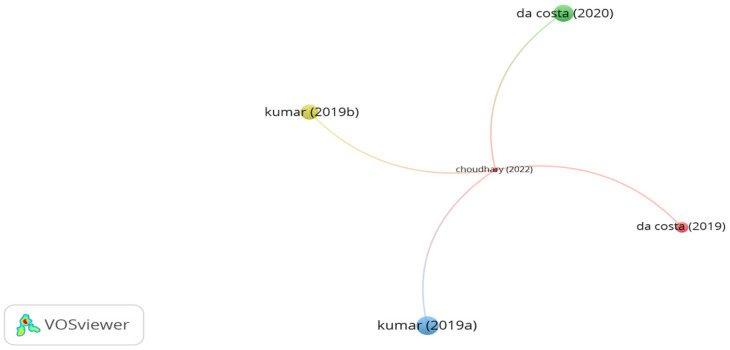
Network visualization of banana fibers.

The use of banana fibers as reinforcement materials in asphalt mixtures has increased in the last 3 years. Significant studies utilizing banana fibers in asphalt mixtures are highlighted in Figure 19. The significant studies were identified through citation analysis. Kumar et al. [167,176] concluded that amending SMA with 0.3% banana fibers by mix mass enhanced the rutting and cracking resistance and reduced drain down of SMA. Costa et al. [177] studied the performance of modified stone mastic asphalt at various lengths and proportions. The superior performance in terms of indirect tensile strength, resilient modulus, dynamic modulus, and drain down was observed by adding 20 mm long banana fibers at a proportion of 0.3% by SMA weight. In another study conducted by Costa et al. [178], the addition of 20 mm long banana fibers at a dosage of 0.3% by mix weight into SMA enhanced Marshall stability, modified Lottman, indirect tensile strength (ITS), and Cantabro. 

### 5.4. Applications of Waste Fibers in Asphalt Mixes 

#### 5.4.1. Polyethylene Terephthalate (PET) Fibers

**Figure 20:**
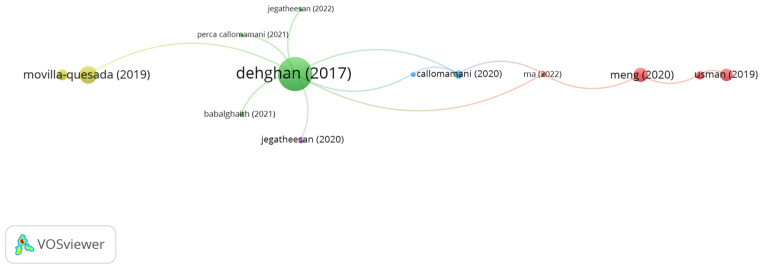
Network visualization of PET fibers.

PET fibers are produced from waste plastic bottles. The global increase in the waste of plastic bottles prompted scientists and engineers to use these wastes in civil engineering applications [179]. Recently, the use of PET fibers in asphalt mixes remarkably increased, in particular in the last three years. Significant studies using PET in asphalt applications have been reviewed based on the citation analysis. Figure 20 demonstrates the network map of influential studies. It is seen in the figure that the number of clusters is 5, while the number of links is 17. However, the document citations number of Dehghan, Usman, Movilla-Quesada, Jegatheesan, and Babalghaith was 48, 7, 13, 0, and 1, respectively. Dehghan et al. [179] stated that adding 20 mm long PET fibers by a dry process at a dosage of 1% and crumb PET at a content of 2% by binder weight significantly improved fatigue cracking by 148% and 163%, respectively, as compared to the control mix. Usman et al. [180] investigated the potential use of 10 mm long PET fibers in hot mix asphalt. PET fibers were introduced to the mix at proportions of 0.3, 0.5, 0.7, and 1% by mix weight. The authors noticed that adding PET fibers at 0.5% showed the best performance regarding rutting resistance and resilient modulus. Movilla-Quesada et al. [181] concluded that amending bitumen with 30–50 mm long PET fibers at doses of 10% and 12% by bitumen weight enhanced the physical properties of the binder in terms of penetration, consistency, ductility, and viscosity. According to Jegatheesan et al. [182,183], modifying bitumen with 4–6 mm long PET fibers at a content of 10% by weight of bitumen improved the physical properties of the binder and the performance of the asphalt mix composed of 18% carbonized wood as fine aggregates in terms of Marshall stability, resilient modulus, and indirect tensile strength. Babalghaith et al. [184] indicated that adding 4 mm long black PET fibers to SMA at a content of 0.6% by mix weight significantly enhanced the stiffness modulus and indirect tensile strength of SMA.

#### 5.4.2. Tire Textile Fibers

**Figure 21:**
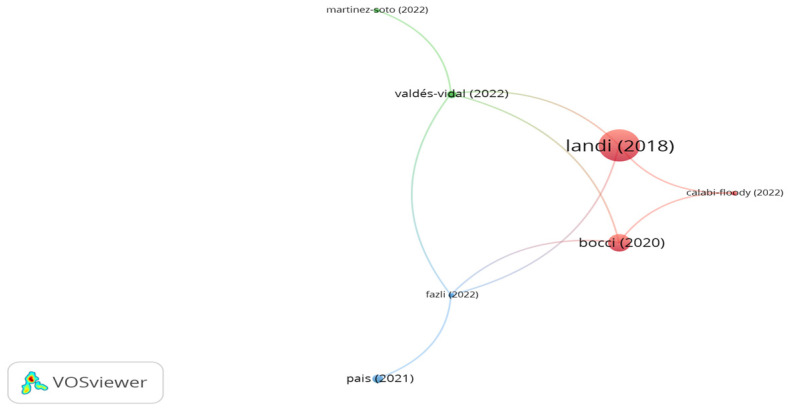
Network visualization of tire textile fibers.

Tire textile fibers represent 10–15% of end-of-life tire weight. End-of-life tires have become a major environmental challenge due to the annual constant increase in these wastes. As a result, utilizing tire textiles as reinforcement materials in asphalt mixes is a successful approach in terms of decreasing the quantities of end-of-life tires [185]. Figure 21 summarizes the influential studies utilizing tire textile fibers in asphalt mixes. As seen in the figure, the number of clusters is 3, while the links number is 9. The documents of Landi, Bocci, and Valdés-Vidal represents the significant clusters. However, the number of citations for the document of Landi, Bocci, Valdés-Vidal, and Calabi-Floody was 50, 15, 3, and 0, respectively. Landi et al. [141,142] confirmed that the addition of 0.3% end-of-life tires to a porous asphalt mix significantly improved the fatigue life of the asphalt mix. Bocci et al. [185] concluded that reinforcing hot mix asphalt with 1–2.5 mm long tire textile fibers at a content of 0.3% by mix weight enhanced the fatigue cracking resistance of the asphalt mix. However, the stiffness modulus and indirect tensile strength of the reinforced asphalt mix were almost similar to the control mix. Additionally, the authors stated that a filler with a content of 2% should be added due to the decrease in the compactability of the reinforced asphalt mix. Valdés-Vidal et al. [186] studied amending a binder with tire textile fibers at different percentages of 2–8% in 3% increments by bitumen weight. The researchers found that adding fibers at the percentages of 2% and 5% significantly enhanced the stiffness modulus, permeant deformation, and cracking resistance at intermediate and low temperatures of the asphalt mix. Furthermore, the authors noted that the addition of fibers requires a higher compaction level to achieve the required density. Calabi-Floody et al. [187] detected that adding 0.5% tire textile fibers as a bitumen modifier decreased the permanent deformation of the binder. In addition, the researchers confirmed that there was no negative impact on the performance of the binder at low temperatures. 

#### 5.4.3. Metallic Fibers

**Figure 22:**
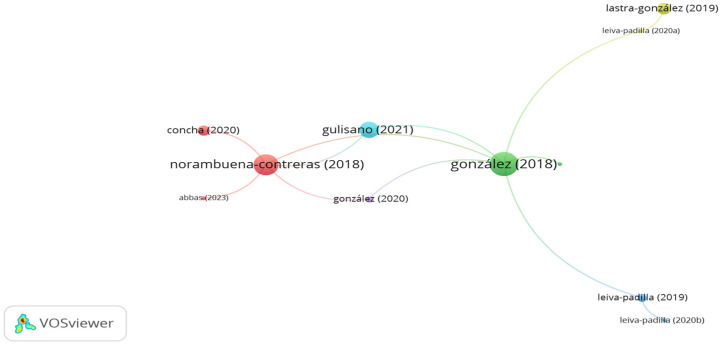
Network visualization of metallic fibers.

Metallic fibers are common waste materials produced from waste tires. Lately, the use of metallic fibers in asphalt mixes has increased as a contribution towards sustainable practices. In accordance with the outputs of citation analysis, the network map of the significant studies using metallic fibers in asphalt mixes is shown in Figure 22. As seen in the figure, the document of González and Norambuena-Contrera showed the highest number of citations. However, the citations for document of González and Norambuena-Contrera were 56 and 45, respectively. González et al. [188] assessed the performance of hot mix asphalt incorporating amended bitumen with different proportions of metallic fibers of 1.5, 2.5, and 3.5% by bitumen volume. The findings of the study demonstrated that the higher the dosage of fiber, the higher the air void content, clustering, and electrical conductivity. Additionally, the authors stated that fibers decreased the stiffness modulus and indirect tensile strength, while the tensile strength ratio was above 80%. In another study conducted by González et al. [189], the feasible use of reclaimed asphalt pavement (RAP) in an asphalt mix modified with metallic fibers by the wet process was assessed. The study findings illustrated that the integration of RAP increased the stiffness modulus and tensile strength of the mix, while metallic fibers improved cracking healing through microwave heating. Similarly, Norambuena-Contrera et al. [109] concluded that the incorporation of metallic fibers increased air voids and thermal conductivity, while the distribution of fibers was not uniform. Table 3 summarizes the findings of the review studies.

### 5.5. Effect of Adding Fibers on the Volumetric Properties of Asphalt Mix

Based on the previous studies, Table 4 demonstrates the impact of fibers on the volumetric properties of an asphalt mix. As can be seen in the table, the addition of fibers decreases the specific gravity of the asphalt mix as compared to the control mix. The higher the content of fibers, the lower the density of the asphalt mix. This is attributed to the lightweight fibers. Furthermore, it is observed in the table that introducing fibers to an asphalt mix at the optimum bitumen content of the control mix notably increases the air void content (AV) due to the absorption property of some fibers. The increase in the absorption rate of fibers results in higher air void content. Thus, the addition of bitumen is required to meet the specification of air void content. Furthermore, the voids in mineral aggregates (VMA) refer to the volume of unabsorbed bitumen by aggregates and air void content in an asphalt mix. VMA is a critical property because it has a direct effect on the performance of an asphalt mix. The higher the VMA content, the thicker the film thickness, and the better the resistance to aging and moisture susceptibility. Moreover, it is displayed in Table 4 that the incorporation of fibers in an asphalt mix results in a higher VMA content as compared to an unreinforced mix. The higher the content of fibers, the higher the VMA. This is attributed to the slight reduction in the specific gravity of asphalt mixes integrated with fibers and the increase in bitumen content. Furthermore, voids filled with asphalt (VFA) refer to the voids filled with a binder in the compacted sample. VFA depends on VMA and AV, and as the percentage of VMA increases and the percentage of AV decreases, the percentage of VFA increases [67,86,124,146].

## 6. Research Gaps and Recommendations

The use of natural and synthetic fibers in asphalt applications is increasing remarkably, in particular in the last five years. However, there are gaps in the research literature that need to be addressed. There is a lack of information that describes the performance of reinforced asphalt mixtures subjected to long-term aging. Additionally, the biodegradability of natural fibers and its impact on the performance of asphalt mixtures are not addressed. Furthermore, the period of natural fibers’ decomposition in asphalt mixtures is not evaluated in the research literature. The effect of the adsorption of bitumen oil and light components by fibers on the aging resistance of asphalt mixes is not addressed well. The behavior and performance of elastic materials depend on the type of applied load stress/strain. Accordingly, the elastic and ductile behavior of a fiber-modified asphalt mix under different strains and stress levels is not addressed well. The low-temperature performance of SMA and porous asphalt mix incorporated with natural fibers is not highlighted well. Furthermore, the influence of coarser/finer gradation of dense asphalt mixtures on the performance of fiber-modified asphalt mixes needs to be assessed.

In accordance with the gaps mentioned above, the following recommendations are proposed.

- The performance of fiber-reinforced asphalt mixes subjected to long-term aging should be evaluated.
- The decomposition period of natural fibers in asphalt mixtures should be identified.
- The influence of load type stress/strain on the performance of reinforced asphalt mixes should be assessed.
- The low-temperature performance of fiber-modified SMA/porous asphalt mix should be highlighted.
- The gradation impact on the performance of asphalt mixes incorporated with fibers should be investigated.
- The integration of natural and synthetic fibers in asphalt mixtures should be evaluated.
- Effect of short-term aging and long-term aging on the thermal degradation of fiber-modified bitumen should be studied.
- Performance of asphalt mix incorporated natural fibers with treated surface should be highlighted.
- The addition of natural/synthetic fibers to polymer-modified bitumen should be assessed.

## 7. Conclusions

This paper aimed to review the most influential studies using synthetic, natural, and waste fibers in asphalt mixes. The conclusions can be stated as follows:

- Based on the annual publications, there is a growing interest in the incorporation of natural and waste fibers in asphalt mixes.
- Modifying bitumen with basalt, carbon, polyester, cellulose, lignin, PAN, polypropylene, palm, and coconut significantly enhanced the physical and rheological properties of the base binder.
- The most used dosage and length of fibers in asphalt mixes are 0.3% by aggregates/ mix weight and 6 mm, while a proportion of 5% by bitumen weight is used for modifying bitumen.
- The incorporation of fibers in asphalt mixes remarkably improved the low-temperature and high-temperature performance of asphalt mixes.
- Introducing carbon and steel fibers enhances the conductive property of asphalt mixes.
- Utilizing natural fibers in SMA and porous asphalt mixes notably reduces drain down and improves the mechanical performance of asphalt mixes. However, adding fibers to porous asphalt mixes decreases the air void content and permeability.
- Adding fibers into asphalt mixes incorporated with reclaimed asphalt pavement significantly improves the performance of asphalt mixes.
- Introducing fibers to asphalt mixes has a significant impact on the volumetric properties of the mixes in terms of bitumen content, density, VMA, and VFA.

## Figures and Tables

**Table 1:** Physical, mechanical, and thermal properties of fibers [39,40,41,42,43,44,45].

	Physical Properties		Mechanical Properties			Thermal Properties		
Fiber	Density (g/cm^3^)	Moisture Content (%)	Tensile Strength (MPa)	Young’s Modulus (GPa)	Elongation (%)	Thermal Conductivity (W/m.K)	Glass Transition (Tg) (°C)	Melting Point (Tm) (°C)
Natural fibers								
Bamboo [41]	0.6–1.1	8.9	140–230	11–17	-	-	-	-
Palm [41,44]	0.7–1.55	-	248	3.2	25	0.199	-	-
Coconut [40,44]	1.15–1.46	8	95–230	2.8–6	15–51.5	0.047	-	-
Sisal [40,44]	1.33–1.5	10–22	363–700	9–38	2–7	0.042	-	-
Kenaf [40]	1.40	9–12	223–930	14.5–53	1.5–2.7	-	-	-
Banana [39]	1.40	8.7–12	529–914	27–32	3	-	-	-
Jute [41]	1.30–1.40	12.60	393–773	13–26.50	1.2–1.5	-	-	-
Synthetic fibers								
Polypropylene [43,45]	0.91	-	500–700	3.5–6.8	21	0.12	−20 to −50	165
Polyester [43,45]	1.38	-	400–600	8.4–16	11-30	0.13	64	240
Basalt [43]	2.6–2.7	-	3100–4800	85–95	3.1	-	-	1450
Carbon [42]	1.8-1.9	-	1700–2600	140–200	0.8–1.5	8–70	-	3500
Glass [42]	2.5–2.56	-	1700–3500	27	2.5–3.2	0.04	-	1540
Aramid [41,44]	1.4	-	3000–3150	63–67	3.3–3.7	0.05	-	500
PAN [45]	1.17	-	200–400	20	27–48	-	97	330
Steel [43]	7.85	-	400–1200	200	3.5	50	-	800

**Table 2:** Degradation periods of natural fibers [46].

Fiber	Period (Days)
Bamboo	1–120
Oil palm	21–90
Sisal	21–90
Banana	28–90
Jute	21–35
Kenaf	30–180
Coconut	21–60
Cotton	21–28

**Table 3:** Summary of findings.

Citations	Fiber	Content (wt%)	Length (mm)	Penetration (%)	Softening Point (%)	Viscosity (%)	Ductility (%)	Rutting (%)	Fatigue (%)	Shear Strength (%)
Fiber-modified bitumen										
[60]	Polyester	5	6	393				90		↑
[60]	Basalt	7	6	394				285		↑
[160]	Basalt	3	6		12		11	90		
[96]	Glass	1 (vl%)	6	89	40	500		20		
[130]	PAN	3	6					8		
[133]	PAN	1	6							
[135]	Ceramic	3	20	50	20	33	690	55	35	
[154]	Carbon	2.75	0.15	335	16	400		8		
[126]	Aramid	2	2	10	33		≅	3		
[154]	Palm	0.75	<0.58	58	8	900		5		
[60]	Lignin	3	<3	77				329		310
[160]	Lignin	3	<3	≅			60	10		
[162]	Coconut	6	2.5					50		
[96]	cellulose	1 (vl%)		13	31	400		30		
Fiber-modified dense asphalt mix										
Citations	Fiber	Content (wt%)	Length (mm)	Stability (%)	TSR (%)	Rutting (%)	Fatigue (%)	Low temperature (%)		Fracture energy (%)
[61]	polypropylene	0.5 (vl%)	6	13	50	40		60 (−10 °C)		
[64]	Polyester	0.25	8					37 (−18 °C)		23 (25 °C)
[94]	Polyester	0.4	25			15	≅			
[69]	Polyester	0.40	12							28 (0 °C)
[61]	Polyester	1 (vl%)	6	15	4	30		32 (−10 °C)		
[70]	Polyester	0.3	6		13	10	28			
[74]	Basalt	0.5	6					300 (−10 °C)		350 (−10 °C)
[84]	Basalt	0.4	9					40 (−20 °C)		
[70]	Basalt	0.3	6		12	23	33			
[74]	Glass	0.5	6					60 (−10 °C)		210 (−10 °C)
[87]	Glass	0.12	12					10 (−15 °C )		50 (0 °C)
[91]	Glass	0.3	12		12			19 (−10 °C)		
[94]	Glass	0.4	36			90	20 (10 °C)			
[111]	Carbon	0.3	4					20 (−10 °C)		
[61]	Carbon	1 (vl%)	6	≅	40	≅		15 (−10 °C)		
[74]	Steel	0.5	6					40 (−10 °C)		90 (−10 °C)
[122]	polypropylene-aramid	0.3	19					26 (−15 °C)		7 (15 °C)
[122]	PAN	0.3	4					22 (−15 °C)		13 (15 °C)
[123]	PAN	0.2	6		↓					79
[138]	Ceramic	0.4	2–4	18	6	23		10 (−10 °C)		
[145]	Bamboo	0.2	6	9	10	30		22 (−10 °C)		
[146]	Bamboo	0.35	<6	16	9	67		12 (−10 °C)		
[91]	Lignin	0.3	1.1		6			20 (−10 °C)		
[70]	Lignin	0.3	<5		320	15	19			
[146]	Lignin	0.4	<5	14	7	58		1.09 (−10 °C)		
[161]	Coconut	0.4	10	15						
[166]	Sisal	0.3	10	13	12					
[111,169]	Kenaf	0.3	8					1.15 (−15 °C)		
[170]	Kenaf	0.3	30	3	13	50	↑			
[179]	PET	0.5	20			↑	↑			
[180]	PET	0.5	10							
[185]	Tire textile	0.3	1–2.5			↑	↑			
Fiber-modified stone mastic asphalt										
Citations	Fiber	Content (wt%)	Length (mm)	Stability (%)	Stiffness modulus (%)	ITS (%)	TSR (%)	Rutting (%)	Low temperature (%)	Drain down
[158]	Basalt	0.4	6					25	10 (−10 °C)	
[147]	Bamboo	0.4	<6	11				6	11 (−10 °C)	
[152]	Palm	0.3	5–25	18	30	23	12			↓
[167]	Coconut	0.3	0.1–1.5			38	88			
[167]	Sisal					35	86			
[166]	Sisal	0.3	10	16		16	9			↓
[171]	Kenaf	0.2	10	7	≅			↓		
[115]	Jute	0.5	7.5	19				1.5		↓
[115]	Carbon	0.5	7.5	30				2.1		↓
[178]	Banana	0.3	20		↓	50		2.2	↑	↓
Fiber-modified porous asphalt mix										
Citations	Fiber	Content (wt%)	Length (mm)	Cantabro (%)	Stiffness modulus (%)	Air voids/permeability	ITS/TSR	Rutting (%)	Fatigue (%)	Drain down
[62]	Polyester	0.3	6	500	↓			2	↑	↓
[73]	Basalt	0.2	24	18		↓	16/0.9			
[79]	Basalt	0.3	9	85			30/2	52	40	↓
[62]	Basalt	0.15	6	500	≅			200	↑	↓
[73]	Glass	0.2	12	227		↓	43/5			↓
[62]	PAN	0.15	6	490	↓			60	↑	↓
[125]	Aramid	0.05	12	7		↓	5/0.7			
[62]	Lignin	0.3	<6	95	↓			200	↑	↓
[164,165]	Coconut	0.3	-			↓		12		↓

↑ Increase of the parameter; ↓ decrease of the parameter; ≅ results similar to control.

**Table 4:** Influence of adding fibers on the volumetric properties of asphalt mix.

Fiber	Fiber Content (%)	Bitumen Content (%)	AV (%)	VMA (%)	VFA (%)	Gmb (g/cm^3^)
Control [86]	0	5.6	3.3	16.80	80.35	2.593
Basalt	0.15	5.80	3.70	17.60	78.98	2.573
	0.30	6.20	3.80	19.20	80.20	2.537
Glass	0.15	5.60	3.70	17.10	78.36	2.584
	0.30	6.20	3.40	18.50	81.62	2.560
Carbon	0.15	5.60	3.70	17.10	78.36	2.584
	0.30	6.40	3.60	19.30	81.35	2.541
Control [145]	0	4.85	4.30	15.00	71.70	-
Lignin	0.30	5.10	4.30	16.00	72.70	-
Polyester	0.20	4.90	4.20	15.20	72.40	-
Bamboo	0.30	5.30	4.00	15.90	74.80	-
Control [123]	0	5.50	4.00	14.90	73.80	2.337
PAN	0.30	5.50	6.43	-	-	2.270
	0.30	6.00	5.25	-	-	2.130
PET	0.30	5.50	6.81	-	-	2.300
	0.30	6.00	4.79	-	-	2.310
Control [67]	0	4.90	4.00	16.00	75.00	2.560
PVA	0.30	5.10	4.00	16.20	75.30	2.539
Acrylic	0.30	5.20	4.00	16.53	75.80	2.536
Polyester	0.30	5.20	4.00	16.13	75.20	2.541

## Data Availability

The presented data are available in the article.
